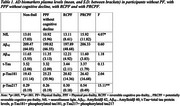# AD biomarkers in cognitive pre‐frailty: Preliminar results from the CompAS

**DOI:** 10.1002/alz70857_105031

**Published:** 2025-12-24

**Authors:** David Facal, Ana I. Rodríguez‐Pérez, Fátima Fernández‐Feijoo, Sonali Arora, Sabela C. Mallo, Juan J. Ansede‐Bermejo, Onésimo Juncos‐Rabadán, Arturo X. Pereiro Rozas

**Affiliations:** ^1^ Instituto de Psicoloxía (IPsiUS), Universidade de Santiago de Compostela, Santiago de Compostela, Galicia, Spain; ^2^ Departamento de Psicoloxía Evolutiva e da Educación, Universidade de Santiago de Compostela, Santiago de Compostela, Galicia, Spain; ^3^ Instituto de Investigación Sanitaria de Santiago de Compostela (IDIS), Santiago de Compostela, Galicia, Spain; ^4^ Centro Singular de Investigación en Medicina Molecular e Enfermidades Cronicas (CIMUS), Universidade de Santiago de Compostela, Santiago de Compostela, Galicia, Spain; ^5^ Centro de investigación Biomédica en Red sobre Enfermedades Neurodegenrativas (CIBERNED), Santiago de Compostela, Gallicia, Spain; ^6^ Centro Nacional de Genotipado (CEGEN‐PRB3‐ISCIII), Universidade de Santiago de Compostela, Santiago de Compostela, Galicia, Spain

## Abstract

**Background:**

Cognitive frailty (CF) is defined as the simultaneous presence of physical frailty (PF) and cognitive impairment in the absence of dementia or other brain disorders. CF is considered reversible in the presence of physical pre‐frailty (PPF) and Subjective Cognitive Decline (SCD), and potentially reversible in the presence of PF and Mild Cognitive Impairment (MCI). Previous studies have related CF with neuroimaging biomarkers (Facal et al., 2021), however the relationship between FC and plasma biomarkers specific to AD, such as NfL, Ab_42_, Ab_40_, t‐Tau, *p*‐tau181 and *p*‐tau217, has been less studied.

**Method:**

The sample consisted of 325 participants from the CompAS who successfully completed cognitive and functional assessment and plasma analysis. Of these, 78 (24%) were non‐frail, 66 (20.3%) had PPF without cognitive decline, 84 (25.8%) reversible cognitive pre‐frailty (RCPF), 81 (24.9%) potentially reversible cognitive pre‐frailty (PRCPF), 2 (0.6%) PF without cognitive decline, 7 (2.2%) reversible CF, and 7 (2.2%) potentially reversible CF. Participants in the PF without cognitive impairment, reversible PF, and potentially reversible PF groups were rare and were excluded from the analyses.

The concentrations (pg/mL) of NfL, Aβ‐40, Aβ‐42, tau, *p*‐tau217, and *p*‐tau181 were quantified using the ultra‐sensitive Single Molecule Array (SIMOA) technology on the Simoa SR‐X platform (Quanterix). The corresponding commercial kits (PCs. 104073, 101995, 104570, and 104111) were used in strict accordance with the manufacturer's protocol. Group‐comparisons were carried out considering non‐frail, PPF without cognitive decline, RCPF and PRCPF participants.

**Results:**

Significant differences were found in NfL and *p*‐Tau217 (Table 1), with lower levels in the group of PPF without cognitive decline compared with the group with PRCPF (Bonferroni test *p* < 0.05).

**Conclusion:**

The differences between the group of PPF without cognitive decline and the group with PRCPF seem to indicate that the presence of plasma AD markers is higher related to the cognitive status of the participants than to their functional status. Further investigation of this relationship would require regression models with CF status as the dependent variable and plasma AD biomarkers and functional markers as predictors.